# A Longitudinal Study of Association between Heavy Metals and Itchy Eyes, Coughing in Chronic Cough Patients: Related with Non-Immunoglobulin E Mediated Mechanism

**DOI:** 10.3390/ijerph13010110

**Published:** 2016-01-07

**Authors:** Thao Thi Thu Nguyen, Tomomi Higashi, Yasuhiro Kambayashi, Enoch Olando Anyenda, Yoshimasa Michigami, Johsuke Hara, Masaki Fujimura, Hiromasa Tsujiguchi, Masami Kitaoka, Hiroki Asakura, Daisuke Hori, Yuri Hibino, Tadashi Konoshita, Hiroyuki Nakamura

**Affiliations:** 1 Department of Environmental Health and Preventive Medicine, Graduate School of Medical Sciences, Kanazawa University, 13-1 Takara-machi, Kanazawa 920-8640, Japan; toi_fs@yahoo.com (T.T.T.N.); ykamba@med.kanazawa-u.ac.jp (Y.K.); enolany@yahoo.com (E.O.A.); t-hiromasa@med.kanazawa-u.ac.jp (H.T.); m-ymzk16@stu.kanazawa-u.ac.jp (M.K.); heros@stu.kanazawa-u.ac.jp (H.A.); hori_d@mbr.nifty.com (D.H.); hibino@staff.kanazawa-u.ac.jp (Y.H.); 2 Department of Hygiene, Graduate School of Medical Sciences, Kanazawa University, 13-1 Takara-machi, Kanazawa 920-8640, Japan; tomotomo@med.kanazawa-u.ac.jp; 3 Environment Preservation Center, Kanazawa University, Kakuma-machi, Kanazawa 920-1192, Japan; mitigami@t.kanazawa-u.ac.jp; 4 Respiratory Medicine, Cellular Transplantation Biology, Graduate School of Medical Sciences, Kanazawa University, 13-1 Takara-machi, Kanazawa 920-8640, Japan; hara0728@gmail.com (J.H.); fujimura@nanao.hosp.go.jp (M.F.); 5 Respiratory Medicine, National Hospital Organization Nanao Hospital, 3-1 Mattou-machi Yabe, Nanao, Ishikawa 926-8531, Japan; 6 Third Department of Internal Medicine, Fukui University School of Medicine, Eiheiji, Fukui 910-1193, Japan; konosita@u-fukui.ac.jp

**Keywords:** heavy metals, coughing, itchy eyes, chronic cough, total serum IgE level

## Abstract

The association between heavy metals exposure and respiratory diseases or allergic sensitization showing high serum immunoglobulin E (IgE) has been suggested. However, previous findings have been inconsistent and the mechanisms responsible remain unclear. We evaluated heavy metal exposure and its association with coughing, itchy eyes in chronic cough patients with different IgE levels. Ninety outpatients in Kanazawa University Hospital were recruited between January–June 2011. Subjects whose total IgE measured by radioimmunosorbent test were asked to record their daily symptoms. We collected daily total suspended particles (TSP) from which concentrations of calcium (Ca), cadmium (Cd), chromium (Cr), iron (Fe), manganese (Mn), nickel (Ni), and lead (Pb) were determined then divided into high and low level groups. Generalized estimating equations were applied to compute the relationship between concentrations of these metals and symptoms. All metals at high levels were significantly associated with itchy eyes compared with low levels, with exception of Ca, the six others were significant in patients with IgE < 250 IU/mL. Cd, Fe, Mn had association with coughing (odds ratio-OR (95% confidence interval-CI): 1.13 (1.03, 1.24), 1.22 (1.05, 1.42), and 1.13 (1.01, 1.27), respectively), this relationship remained significant for Cd (OR (95% CI): 1.14 (1.03, 1.27)) and Mn (OR (95% CI): 1.15 (1.00, 1.31)) in patients with lower IgE. Our findings demonstrate the relationship between aerial heavy metals and itchy eyes, coughing in chronic cough patients, suggesting these symptoms may be due to a non-IgE mediated mechanism.

## 1. Introduction

Due to economic and industrial growth, air pollution such as particulate matter (PM) and gaseous emissions have become an important environmental issue throughout the world. PM is a heterogeneous mixture of biologically active components in the air, and heavy metals and polycyclic aromatic hydrocarbons have been identified as the predominate toxic constituents [1,2,3]. Airborne heavy metals originate from various sources involving diesel exhaust emissions (iron (Fe), manganese (Mn), lead (Pb), cadmium (Cd), calcium (Ca), and nickel (Ni)) and non-exhaust emissions such as the abrasion of brakes and tires (Fe and copper (Cu)), and are regarded as urban air pollutants that have negative effects on health [4,5].

Epidemiological and experimental studies have provided evidence for the adverse effects of PM exposure, particularly heavy metals, on respiratory diseases and allergic sensitization. For example, exposure to ambient PM has been identified as a high risk factor for increasing the incidence of atopic diseases and allergic sensitization in children [6]. Heavy traffic exposure, one source of heavy metals, has associated with increases in the incidence and prevalence of childhood asthma and wheeze as well as allergic sensitization, bronchial hyperresponsiveness and respiratory symptoms in children [7,8]. A previous study reported that the concentration of Fe in PM_10_ was associated with allergic sensitization at the birth address as well as the current address and may also increase the risk of asthma and allergy in schoolchildren [9]. Increases in ambient Ni concentrations significantly increased wheeze symptoms in young children in New York City [10]. Higher levels of Fe and Cd have been detected in the exhaled breath condensates of chronic obstructive pulmonary disease patients than in those of non-smoking subjects [11]. However, this relationship is weak or inconsistent and the underlying mechanisms for the adverse effects of pollutants on human health currently remain unclear.

Chronic cough is the fifth most common condition encountered by primary care physicians [12]. In addition to eosinophilic bronchitis, gastro-esophageal reflux disease, postnasal drip syndrome, and rhinosinusitis, chronic obstructive pulmonary disease, pulmonary fibrosis, bronchiectasis, and bronchial asthma have been identified as some of the most common causes of chronic cough worldwide. Cough-variant asthma and atopic cough, two asthma-related conditions, also accompany chronic cough [13]. In Japan, coughing is the most common reason for patients (11.7% of all) to visit clinics [14], and cough-variant asthma as well as atopic cough have been identified as the main causes of isolated chronic non-productive cough [15]. Previous studies in the United Kingdom and Australia identified the most common cause of coughing as rhinitis, followed by asthma [16,17].

Immunoglobulin E (IgE) plays an important role in inflammation. It has interdependent and independent roles in the complex immune responses that manifest clinically as allergic disorders [18]. Inhaled environmental exposure and its influences on T-helper (Th) cell and serum IgE have been demonstrated [19,20]. Possible mechanism of the effects of heavy metals on allergic inflammation has been suggested in which heavy metals suppress Th1 development by inhibiting interferon-γ (IFN-γ) expression and promote Th2 development by enhancing interleukin 4 expression, increase IgE as well as IgE-dependent basophil-mediated inflammation [21]. Several experimental studies on heavy metals have implicated Fe exposure in alterations in humoral and cell-mediated immunity and the development of allergic conditions, including the production of serum IgE [22,23], and Cd at low concentrations has been suggested to inhibit the synthesis of IgE by human B lymphocytes [24]. In contrast, an epidemiological study on an urban Canadian population of pregnant woman and their children found no relationship between metals and elevated serum IgE [25]. Furthermore, there is currently no evidence for average total serum IgE levels in patients with chronic respiratory diseases and allergic sensitization whose total serum IgE levels may be normal or high. Hence the effects of heavy metals on IgE-mediated allergic responses as well as the roles of IgE in airway inflammation have not yet been elucidated in detail.

Information provided by longitudinal studies on the relationship between heavy metal exposure and respiratory diseases is still limited. The involvement of IgE mechanisms in the symptoms of patients with chronic airway inflammation remains unclear. Therefore, we conducted this study to investigate the relationship between heavy metal concentrations and the appearance of symptoms including itchy eyes and coughing in chronic cough patients with different total serum IgE levels.

## 2. Experimental Section 

### 2.1. Participant Recruitment

**Figure 1:**
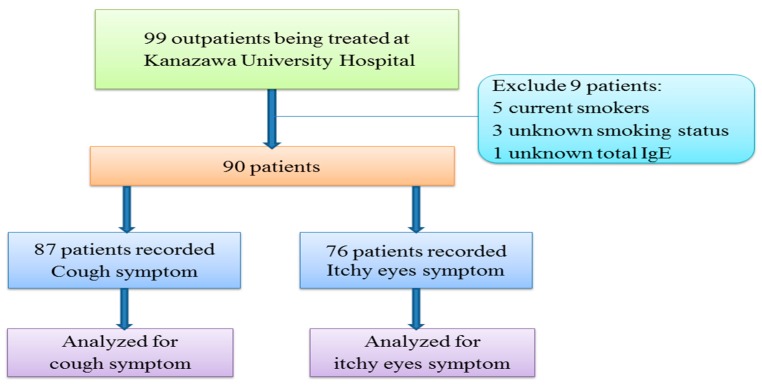
Flow chart showing description of patients in the study.

Ninety-nine patients diagnosed with at least one of the following: Bronchial asthma, atopic cough, and cough-variant asthma, were recruited at Kanazawa University Hospital between 4 January 2011 and 30 June 2011. All subjects gave their informed consent for inclusion before they participated in the study. The study was conducted in accordance with the principles of the Declaration of Helsinki of the World Medical Association, and the protocol was approved by the Medical Ethics Committee of Kanazawa University (Project Identification Code: 981). We used Japan Asthma Prevention and Management Guidelines 2011 to diagnose asthma [26]. The criteria of the Japanese Cough Research Society was used to diagnose cough-variant asthma [27]. Atopic cough was diagnosed using reported criteria, which are based on bronchodilator-resistant cough and the resolution of coughing with the use of histamine H1 antagonists and/or inhaled corticosteroids (ICS) [28]. Detailed recruiting methods have been published elsewhere [29]. We excluded five current smokers, three patients with an unknown smoking status, and one patient whose total serum IgE level was not obtained. Among the remaining 90 patients, 87 had a recorded frequency of coughing, while 76 had a recorded level of itchy eyes (Figure 1).

Among the 76 patients with recorded itchy eye symptoms, 59 had a total serum IgE level less than 250 IU/mL, while that of 17 patients was more than or equal to 250 IU/mL (Table 1). Forty-three, 18, and 29 patients were diagnosed with bronchial asthma, cough-variant asthma, and atopic cough, respectively. Among the 87 patients with recorded cough symptoms, 65 had a total IgE level less than 250 IU/mL, while that of 22 patients was more than or equal to 250 IU/mL. Fifty-four, 18, and 30 patients were diagnosed with bronchial asthma, cough-variant asthma, and atopic cough, respectively. Total serum IgE levels were significantly lower in atopic cough patients than in non-atopic cough patients. No significant differences were observed in gender, age, BMI, smoking status, or the onset or duration of disease between the groups of patients with total IgE ≥ 250 IU/mL and < 250 IU/mL.

**Table 1:** Characteristics of the patients.

Characteristics	Patient-Recorded Itchy Eyes Symptoms (*n* = 76)		Patient-Recorded Cough Symptoms (*n* = 87)	
	IgE < 250 IU/m (*n* = 59)	IgE ≥ 250 IU/mL (*n* = 17)	IgE < 250 IU/mL (*n* = 65)	IgE ≥ 250 IU/Ml (*n* = 22)
Gender (Male/Female)	18/41	13/4	20/45	8/14
Smoking status				
Non-Smoker	44	13	47	14
Ex-Smoker	15	4	18	8
Age (years) (Mean ± SD)	63.8 ± 11.9	56.8 ± 15.4	64.8 ± 11.2	61.6 ± 16.3
BMI (kg/m^2^) (Mean ± SD)	22.9 ± 3.8	23.2 ± 2.6	22.6 ± 3.6	22.8 ± 2.5
Duration of disease (years)	15.0 ± 8.8	18.1 ± 8.1	15.7 ± 10.8	18.8 ± 10.0
Onset (age)	49.0 ± 13.3	38.9 ± 18.8	64.3 ± 11.2	61.1 ± 16.2
BA patients	31	12	37	17
CVA patients	13	5	13	5
AC patients **^a^**,**^b^**	26	3	27	3

SD: standard deviation, BA: Bronchial asthma, CVA: Cough variant asthma, AC: atopic cough. **^a^** significantly different at 0.05 level between two groups of patients with recorded itchy eyes. **^b^** significantly different at 0.05 level between two groups of patients with recorded cough.

### 2.2. Health Outcomes

Patients recorded their symptoms in an allergy diary every morning, afternoon, evening, and night from the beginning to the last day of the study. Patients started recording the frequency of coughing from 4 January 2011, while the level of itchy eyes was started on 1 February 2011. We made every effort to avoid missing data due to patients forgetting to record their symptoms in their diaries by asking them to bring their diaries with them when they came to the hospital to check their symptoms or have consultations. The average frequency of coughing and level of itchy eyes was calculated for each patient. The presence of symptoms was defined based on whether the frequency of coughing or level of itchy eyes of each patient on each day was higher than the average.

### 2.3. Measurement of Total Serum IgE Levels

At the initiation of the study, total serum IgE levels were measured from a blood sample taken from the peripheral blood vessels of all patients using a radioimmunosorbent test. Patients were divided into two groups with total serum IgE < 250 IU/mL and total serum IgE ≥ 250 IU/mL based on criteria used at Kanazawa University Hospital [30].

### 2.4. Measurement of Heavy Metals in Total Suspended Particulates

Hydrochloric acid and nitric acid were purchased from Kanto Chemical Co., Inc. (Tokyo, Japan) for atomic absorption spectrometry. A borosilicate glass fiber filter coated with fluorocarbon (T60A20, 8 × 10 in, Pallflex (Putnam, CT, USA)) was used. Total suspended particulates (TSP) were collected on the borosilicate glass fiber filter coated with fluorocarbon using a high-volume air sampler (120SL, Kimoto Electric Co., Ltd., Osaka, Japan) at a flow rate of 1000 L, located at Kanazawa University (136.7 degrees east longitude, 36.6 degrees north latitude). After 24 h of collecting ambient dust (start at 13:30 to 13:30 on the next day), the collected filter was removed and replaced with a blank filter. The collected filter was kept for 16 h–48 h in a desiccator in order to remove water and then stored at −30 °C before the extraction and analysis of heavy metals. The method of measurement used has been described previously [31]. A quarter of the filter sample was cut into small pieces and placed in a beaker. Fifteen milliliters of hydrochloric acid and 5 mL of nitric acid were added to the filter and heated on a hot plate for 2 h. The supernatant was filtered through filter paper. Five milliliters of hydrochloric acid and 1.5 mL of nitric acid were added to the remaining filter and heated on an electric hot plate for 15 min. The supernatant was filtered through filter paper, added to the filtrated solution, and then evaporated to dryness on the electric hot plate. After cooling, 9 mL of Milli-Q water and 1 mL of nitric acid were added and heated on the electric hot plate in order to dissolve metals for 15 min. After the dilution of metals, Cd, Co, Cr, Cu, Mn, Ni, and Pb were measured using a graphite atomic absorption spectrometer (model: AA-6800, Shimadzu Corporation, Kyoto, Japan), while Ca and Fe were measured using a flame atomic absorption spectrometer (model: AA-7000, Shimadzu Corporation, Kyoto, Japan). Metal data were not obtained between March 2nd and 16th due to the failure of the high-volume air sampler. Cu and Co were excluded from data for statistical analyses due to missing data for more than 20 days.

### 2.5. Statistical Analysis

In order to assess the effects of heavy metals on the appearance of symptoms over the 6-month follow-up, we applied the generalized estimating equation (GEE) model to adjust the relationship between changes in the concentrations of heavy metals and symptoms in the same patient as well as to determine the effects of these metals on the symptoms of patients with different total serum IgE levels. We categorized the concentration of each metal into two levels (high and low levels) using the median of each one. Since patients recorded the frequency of coughing and level of itchy eyes at different periods, we used the median concentration of heavy metals in these periods. The odds ratios of high levels of each metal with 95% confidence intervals were calculated relative to the low levels. The characteristics of patients (BMI, age, and gender), meteorological factors (temperature, humidity, and precipitation), air pollutants (sulfur dioxide (SO_2_)), and pollen count (cedar and cypress pollen) were used as potential confounders. Data were statistically analyzed using the SPSS software program for MS Windows, version 19.0 (SPSS, Inc., New York, NY, USA). The significance of differences was set at *p* < 0.05 for all analyses.

## 3. Results

### 3.1. Heavy Metal Concentrations

During the study period, information on the concentrations of metals was not obtained for 15 days. The range of metal concentrations was very large. The metal with the highest concentration was Fe (mean 503.23 ng/m^3^). The mean Fe concentration was more than 2-fold higher than that of Ca (mean 238.53 ng/m^3^), and at least 13-fold higher than that of Mn (mean 37.54 ng/m^3^) and the others. The average concentrations of metals from high to low were Fe > Ca > Mn > Pb > Ni > Cr > Cd (Table 2).

**Table 2:** Description of heavy metals from 4 January 2011 to 30 June 2011.

Heavy Metals	Days of Observation	Mean	IQR	Minimum	Maximum
Ca (ng/m^3^)	163	238.53 ± 232.06	184.817	41.29	1777.49
Cd (ng/m^3^)	161	0.26 ± 0.42	0.122	0.00	3.44
Cr (ng/m^3^)	163	2.03 ± 2.30	1.279	0.00	17.54
Fe (ng/m^3^)	163	503.23 ± 920.15	247.813	15.50	6926.79
Mn (ng/m^3^)	163	37.54 ± 254.06	8.326	0.00	3211.78
Ni (ng/m^3^)	162	3.31 ± 4.33	1.866	0.00	38.33
Pb (ng/m^3^)	163	5.98 ± 6.25	3.956	0.00	37.33

IQR: interquartile range.

Correlations were observed between most of the metals examined, except for between Mn, Ca, and Cd (Supplementary Table S1). The strongest correlation was observed between Mn and Fe (0.628), whereas the weakest was between Ca and Cd (0.172).

**Figure 2:**
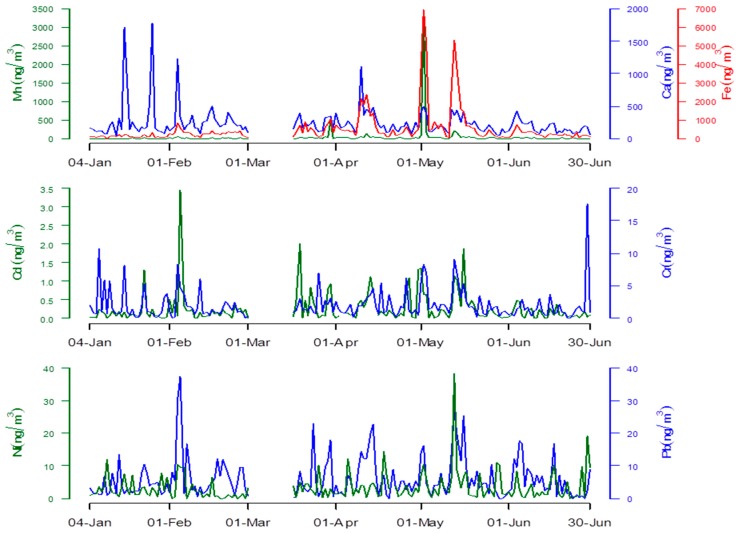
Daily concentrations of heavy metals between 4 January 2011 and 30 June 2011.

Although the concentrations of heavy metals changed every day in a large range (Figure 2), except for Fe, the concentration of which was significantly high in May and Spring, no significant differences were observed between the concentrations of each metal due to the month or season.

### 3.2. Relationship between Heavy Metals and Itchy Eyes and Coughing

Prior to adjusting with potential confounders, no relationship was observed between Ni and itchy eyes. Six other metals at high levels correlated with the appearance of itchy eyes, with ORs for Fe, Mn, Mn, Ca, Cd, Cr, and Pb of 1.83 (95% CI: 1.40, 2.40), 1.77 (95% CI: 1.43, 2.18), 1.49 (95% CI: 1.20, 1.84), 1.34 (95% CI: 1.15, 1.57), 1.29 (95% CI: 1.11, 1.49), and 1.25 (95% CI: 1.05, 1.50), respectively. However, after using covariates (age, BMI, gender, temperature, humidity, precipitation, SO_2_, cedar pollen, and cypress pollen) for adjustments, we found that all metals at high levels correlated with itchy eyes with ORs for Mn, Fe, Cd, Ni, Ca, Pb, and Cr of 1.60 (95% CI: 1.31, 1.95), 1.56 (95% CI: 1.20, 2.02), 1.27 (95% CI: 1.09, 1.47), 1.23 (95% CI: 1.02, 1.49), 1.22 (95% CI: 1.02, 1.45), 1.20 (95% CI: 1.01, 1.41), and 1.17 (95% CI: 1.03, 1.33), respectively (Table 3).

We also evaluated the relationship between heavy metal levels and the occurrence of coughing. Although all metals were positively related to coughing, four out of seven metals correlated with coughing: Fe, Mn, Cd, and Cr with ORs of 1.26 (95% CI: 1.07, 1.48), 1.17 (95% CI: 1.03, 1.33), 1.15 (95% CI: 1.04, 1.26), and 1.12 (95% CI: 1.01, 1.25), respectively. After adjustments for age, BMI, gender, temperature, humidity, precipitation, SO_2_, cedar pollen, and cypress pollen, the relationship between Cr levels and coughing was not significant. However, correlations remained with Fe (OR (95% CI): 1.22 (1.05, 1.42)), Mn (OR (95% CI): 1.13, (1.01, 1.27)), and Cd (OR (95% CI): 1.13 (1.03, 1.24)) (Table 3).

**Table 3:** Adjusted odds ratio and 95% confidence intervals for itchy eyes and cough symptom associated with level of heavy metals’ concentrations.

Metals	Itchy Eyes Symtom				Cough Symptom			
	OR (95% CI) ^a^	*p*-Value ^a^	OR (95% CI) ^b^	*p*-Value ^b^	OR (95% CI) ^a^	*p*-Value ^a^	OR (95% CI) ^b^	*p*-Value ^b^
Ca	1.49 (1.20–1.84)	<0.001	1.22 (1.02–1.45)	0.026	1.11 (0.99–1.25)	0.084	1.03 (0.93–1.13)	0.663
Cd	1.34 (1.15–1.57)	<0.001	1.27 (1.09–1.47)	0.002	1.15 (1.04–1.26)	0.005	1.13 (1.03–1.24)	0.012
Cr	1.29 (1.11–1.49)	0.001	1.17 (1.03–1.33)	0.019	1.12 (1.01–1.25)	0.027	1.10 (0.99–1.02)	0.053
Fe	1.83 (1.40–2.40)	<0.001	1.56 (1.20–2.02)	0.001	1.26 (1.07–1.48)	0.007	1.22 (1.05–1.42)	0.010
Mn	1.77 (1.43–2.18)	<0.001	1.60 (1.31–1.95)	<0.001	1.17 (1.03–1.33)	0.015	1.13 (1.01–1.27)	0.030
Ni	1.18 (0.99–1.41)	0.068	1.23 (1.02–1.49)	0.031	1.01 (0.92–1.11)	0.886	1.02 (0.93–1.13)	0.663
Pb	1.25 (1.05–1.50)	0.015	1.20 (1.01–1.41)	0.038	1.05 (0.97–1.14)	0.227	1.04 (0.96v1.13)	0.376

**^a^** Only heavy metal without adjustment. **^b^** With adjustment for age, BMI, gender, temperature, humidity, precipitation, SO_2_, cedar pollen, cypress pollen.

### 3.3. Relationship between Heavy Metals and Itchy Eyes, Coughing, and Serum IgE Levels

We determined whether symptoms similarly occurred in patients with different total serum IgE levels. Ca was the only metal that did not correlate with the appearance of itchy eyes in patients with total serum IgE levels less than 250 IU/mL. The relationship between heavy metal concentrations and itchy eyes were significant with ORs for Mn, Fe, Cd, Ni, Pb, Cr of 1.65 (95% CI: 1.32, 2.05), 1.61 (95% CI: 1.22, 2.12), 1.30 (95% CI: 1.12, 1.51), 1.23 (95% CI: 1.00, 1.52), 1.21 (95% CI: 1.03, 1.42), and 1.18 (95% CI: 1.03, 1.35), respectively (Table 4). In another analysis, two out of seven metals correlated with the appearance of coughing in patients with total serum IgE levels less than 250 IU/mL, including Mn and Cd with ORs of 1.15 (95% CI: 1.00, 1.31) and 1.14 (95% CI: 1.03, 1.27), respectively. Although Fe also positively correlated with coughing in patients with lower total serum IgE levels (an OR of 1.22 with a *p*-value of 0.027), this relationship did not account for the difference observed in the effects of Fe on coughing in patients with high total IgE levels (an OR of 1.27 and *p*-value > 0.05). No correlations were observed between heavy metal concentrations and symptoms in patients with total serum IgE ≥ 250 IU/mL (Table 4).

**Table 4:** Adjusted odds ratio and 95% confidence intervals for itchy eyes and cough symptom associated with level of heavy metals’ concentration at groups of patients with different total serum IgE levels.

Metals	IgE < 250 IU/mL				IgE ≥ 250 IU/mL			
	Itchy Eyes Symtom		Cough Symptom		Itchy Eyes Symtom		Cough Symptom	
	OR (95% CI)	*p*-Value	OR (95% CI)	*p*-Value	OR (95% CI)	*p*-Value	OR (95% CI)	*p*-Value
Ca	1.16 (0.96–1.40)	0.118	1.06 (0.95–1.19)	0.315	1.56 (0.99–2.47)	0.056	0.91 (0.75–1.12)	0.388
Cd	1.30 (1.12–1.51)	0.001	1.14 (1.03–1.27)	0.014	1.13 (0.72–1.79)	0.599	1.08 (0.88–1.32)	0.458
Cr	1.18 (1.03–1.35)	0.015	1.08 (0.97–1.21)	0.178	1.11 (0.73–1.69)	0.620	1.16 (0.97–1.39)	0.100
Fe	1.61 (1.22–2.12)	0.001	1.22 (1.02–1.45)	0.027	1.34 (0.73–2.48)	0.347	1.27 (0.89–1.81)	0.195
Mn	1.65 (1.32–2.05)	<0.001	1.15 (1.00–1.31)	0.046	1.39 (0.92–2.10)	0.122	1.11 (0.87–1.41)	0.414
Ni	1.23 (1.00–1.52)	0.048	1.03 (0.92–1.17)	0.599	1.21 (0.70–2.07)	0.494	1.00 (0.84–1.20)	0.992
Pb	1.21 (1.03–1.42)	0.019	1.07 (0.97–1.17)	0.194	1.14 (0.64–2.05)	0.657	0.97 (0.81–1.16)	0.703

Adjusted odd ratio and 95% confidence intervals for age, BMI, gender, temperature, humidity, precipitation, SO_2_, cedar pollen, cypress pollen.

## 4. Discussion

To the best of our knowledge, this is the first epidemiological study to provide evidence for a relationship between airborne heavy metals and coughing or itchy eyes in chronic cough patients with different total serum IgE levels. The results of the present study indicate that patients with lower IgE levels are more likely to be affected by heavy metals and also that the relationship between heavy metal exposure and airway inflammation may involve a non-IgE mediated mechanism.

Some environmental studies in large industrial cities in Asia such as Beijing, Guangzhou, Chengdu (China), and Seoul (Korea) reported markedly higher concentrations of PM and heavy metals than those of the present study [32,33,34]. Differences have also been noted in the relationships between metals as well as the order of concentrations reported. These studies showed that the metal with the highest concentration was Pb, an element evoking the greatest concern in terms of environmental heavy metal pollution [35,36], with at least a 2-fold higher concentration than that of Mn, which had the second highest concentration. A recent study showed the metal with the highest concentration was Fe, which has been associated with brake wear in the proximity of busy roads [37], and also that the concentration of Mn was more than 2-fold higher than that of Pb. In addition, a comparison with other studies from similar prefectures located along the coastline facing the Sea of Japan (Tottori) [38] and Pacific Ocean (Nagoya) [39] revealed that the concentrations of heavy metals in Kanazawa in this study followed the same order. These results suggest that the dominant source of metals in Kanazawa, an urban city without large industrial zones is traffic emission from direct tailpipe emission, brake and tire abrasion, and the resuspension of road way dust [40,41].

We clearly showed that all of the metals examined correlated with the presence of itchy eyes after adjusting with covariates. Furthermore, Cd, Cr, Fe, and Mn also correlated with coughing, and this relationship remained significant for Cd, Fe, and Mn after adjusting with possible confounders. A previous study on New York children for 24 months did not find a relationship between coughing and Fe in single models [10]. Other indirect evidence also demonstrated that a higher concentration of transition metals in PM_2.5_ may be responsible for more severe inflammation [42], and short-term reductions in air pollution may improve lung function in children [43]. Children and adolescents with residential proximity to a major roadway more frequently had asthma-related symptoms accompanied by itchy-watery eyes than responders who resided 200–500 meters from a major road [44]. Although Fe was also the main coarse fraction of PM_10_ in the Netherlands with the highest average concentration, a correlation was not observed after adjusting for asthma symptoms and rhinitis but only for allergic sensitization at birth [9]. Exposure to Mn in mine workers has been associated with the appearance of more respiratory symptoms as well as a higher prevalence of impaired lung function [45]. Mn has been suggested as the cause of the high frequency of work-related symptoms such as ocular symptoms and dry cough in welder workers [46]. Ocular surface symptoms have been reported in taxi drivers and traffic controllers due to exposure to air pollution [47]. Our results clearly showed correlations between all heavy metals at high concentrations and itchy eyes, one of the common symptoms of a clinical diagnosis of allergic rhinitis [48], and also between Cd, Fe, and Mn and coughing in multiple-pollutant models. Recent studies have indicated that a relationship exists between asthma as well as chronic respiratory diseases with allergic rhinitis and the recommended reasons of prevalence, links with asthma along with the impact of quality of life and work [49]. The deterioration of upper or lower respiratory, ocular, or skin symptoms has been reported in 33% of asthma patients on Asian Dust day [50]. These findings suggest the presence of a strong relationship between asthma and allergic rhinitis, as supported by our results on metal exposure and the appearance of itchy eyes in patients with respiratory diseases. In the present study, we adjusted for coexisting factors related to allergies such as pollen including Japanese cedar pollen and Japanese cypress pollen. Japanese cedar pollinosis is the most common pollinosis in Japan [51,52], and the prevalence of allergic rhinitis, particularly cedar pollinosis, is increasing [53]. Furthermore, a significantly higher number of patients exhibited itchy eyes and coughing during Asian dust days with pollen [29]. The findings of another study suggest that high levels of anthropogenic air pollution including Fe and Pb are transported with Asian dust [54]. However, it currently remains unclear whether the aggravation of symptoms depends on pollen, metals, or Asian dust. Therefore, we used pollen as a potential confounder in order to better adjust the relationship between heavy metals and symptoms in chronic cough patients.

We clearly demonstrated that correlations existed between six out of the seven heavy metals (except for Ca) at high concentrations and the presence of itchy eyes in patients with total serum IgE levels less than 250 IU/mL. Furthermore, Cd and Mn positively correlated with coughing in patients with total serum IgE levels less than 250 IU/mL. The findings of a current study also showed that none of the metals tested correlated with the appearance of itchy eyes and coughing in patients with total serum IgE levels more than or equal to 250 IU/mL. Several possible mechanisms have been proposed for IgE-dependent allergic inflammation pathology [55,56]. A strong correlation was previously reported between serum IgE levels and asthma and its phenotype [57], suggesting the development of airway hyperresponsiveness and inflammation through the induction of Th2-type cytokine production by IgE in mice [58]. Previous studies have suggested that heavy metals exposure such as lead or cadmium, alters immune system components and is associated with increased production of IgE [59,60,61] However, other studies have suggested effector mechanisms that are independent of IgE may also contribute to the pathology of allergic inflammation. In chronic asthma mouse model, mast cells can substantially influence features of chronic allergic inflammation and tissue remodeling, independently of mast cell signaling through either IgE-FcεRI (high-affinity Fc receptor for IgE) or antigen-IgG-FcγRIII [62]. Different mast cell population show different patterns of expression of receptors for pathogen-associated molecular patterns and activation of mast cells can induce these cells to secrete distinct patterns of cytokines or chemokines [63,64,65,66]. Thus mast cells have potential to drive important features of allergic inflammation independently of IgE. In human, evidence for non-IgE-mediated and irritant-induced work-related rhinitis has recently been increasing [67,68,69]; however, the mechanisms responsible remain largely unclear [69]. Furthermore, Ni has been suggested to act as an allergen that induces an adaptive immune response related to inflammation independently of IgE [55]. Moreover, a previous study on occupational asthma and occupational rhinitis patients caused by new low-molecular-weight molecules including chemicals and metals demonstrated that the prevalence of patients with IgE-mediated mechanisms was markedly lower than the total of patients [70]. These findings suggest the role of non-IgE mediated or other mechanisms in the influence of these factors on asthma and rhinitis patients. In addition, non-IgE-mediated related rhinitis and asthma have recently been described in several case reports, indirect epidemiological studies, and surveillance and experimental research [69]. No epidemiological study has directly shown the effects of total serum IgE levels on the relationship between coughing or itchy eyes and heavy metal concentrations in the air, and the mechanisms underlying IgE-induced airway inflammation still need to be elucidated.

The present longitudinal cohort study with a detailed heavy metal concentration exposure assessment for 6 continuous months with patients recording their symptoms in a diary during the study period in addition to the adjustment of the effects of metals on each patient with possible confounders including patient characteristics (age, gender, and BMI), weather (temperature, humidity, and precipitation), air pollution (SO_2_), and pollen (cedar pollen and cypress pollen) are the important strengths of our study. However, there were some limitations. First, we only obtained data on metal concentrations in TSP at Kanazawa University-Takaramachi Campus; therefore it is possible that data at the observation did not always reflective the concentration in the study area and individual exposures. Second, the findings for metals may be applicable to traffic-related constituents especially largely relate to non-exhaust emission of traffic [9]; however, it is not clear to what extent other sources could be in case metals in the air in some Japan coastal prefectures increase by others events (Asian Dust days) [38,39,54,71]. We only measured the total serum IgE levels of 90 patients at the initiation of the study without re-checking after six months to determine whether changes occurred in order to confirm the relationship between heavy metal concentrations and symptoms related to total IgE levels. Further studies with a larger number of patients whose total serum IgE levels are known are needed in order to more clearly determine the relationship between itchy eyes and coughing in patients with chronic cough and heavy metal concentrations.

## 5. Conclusions

The results of the present study provide evidence for the effects of aerial heavy metals on itchy eyes and coughing in patients with chronic cough, particularly due to different total serum IgE levels. The present results suggest correlations between not only the non-exhaust emissions of traffic and general health, but also asthma and rhinitis. The relationship observed between heavy metal concentrations and the symptoms of our patients with total serum IgE levels less than 250 IU/mL is an important result and indicates the relevance of the impact of heavy metals on airway inflammation through a non-IgE-mediated mechanism.

## Supplementary Files

Supplementary File 1

## Abbreviations

IgE: Immunoglobulin E
TSP: total suspended particles
PM: particulate matter
Ca: calcium
Cd: cadmium
Co: cobalt
Cu: copper
Cr: chromium
Fe: iron
Mn: manganese
Ni: nickel
Pb: lead
BMI: body mass index
SO_2_: sulfur dioxide
OR: Odds ratio
CI: Confidence interval
SD: Standard deviation
SPSS: Statistical Package for Social Sciences
GEE: generalized estimating equation
