# Supplementary material for: A Longitudinal Study of Association between Heavy Metals and Itchy Eyes, Coughing in Chronic Cough Patients: Related with Non-Immunoglobulin E Mediated Mechanism
Source: Int J Environ Res Public Health. 2016 Jan 7;13(1):110. doi: 10.3390/ijerph13010110 (PMC4730501; doi:10.3390/ijerph13010110)
Supplement: Supplementary File 1 [file ijerph-13-00110-s001.pdf]

Supplementary Materials: A Longitudinal Study of Association between Heavy Metals and Itchy Eyes, Coughing in Chronic Cough Patients: Related with Non-Immunoglobulin E Mediated Mechanism

Thao Thi Thu Nguyen, Tomomi Higashi, Yasuhiro Kambayashi, Enoch Olando Anyenda, Yoshimasa Michigami, Johsuke Hara, Masaki Fujimura, Hiromasa Tsujiguchi, Masami Kitaoka, Hiroki Asakura, Daisuke Hori, Yuri Hibino, Tadashi Konoshita and Hiroyuki Nakamura

Table S1. Pearson correlation between daily concentrations of heavy metals.

Metals	Ca (ng/m^3)	Cd (ng/m^3)	Cr (ng/m^3)	Fe (ng/m^3)	Mn (ng/m^3)	Ni (ng/m^3)	Pb (ng/m^3)
Ca (ng/m^3)	1						
Cd (ng/m^3)	0.172 *	1					
Cr (ng/m^3)	0.254 **	0.297 **	1				
Fe (ng/m^3)	0.280 **	0.394 **	0.437 **	1			
Mn (ng/m^3)	0.115	0.124	0.256 **	0.628 **	1		
Ni (ng/m^3)	0.157 *	0.245 **	0.529 **	0.435 **	0.175 *	1	
Pb (ng/m^3)	0.334 **	0.598 **	0.297 **	0.468 **	0.195 *	0.318 **	1

Ca: Calcium, Cd: Cadmium, Cr: Chrome, Fe: Iron, Mn: Manganese, Ni: Nickel, Pb: Lead. * Correlation is significant at the 0.05 level (2-tailed). ** Correlation is significant at the 0.01 level (2-tailed).

© 2016 by the authors; licensee MDPI, Basel, Switzerland. This article is an open access article distributed under the terms and conditions of the Creative Commons by Attribution (CC-BY) license (http://creativecommons.org/licenses/by/4.0/).
